# Non-contrast CT image characteristics on admission predict the 3-month outcome of cerebral venous sinus thrombosis: an observational study in a single institution

**DOI:** 10.1186/s41016-019-0164-9

**Published:** 2019-07-16

**Authors:** Baoqiang Lian, Linsun Dai, Xueling Xie, Dezhi Kang, Guorong Chen, Huangcheng Shangguan, Peisen Yao, Shufa Zheng

**Affiliations:** 1 Department of Neurosurgery, The First Affiliated Hospital of Fujian Medical University, NO. 20 Chazhong Road, Taijiang District, Fuzhou, 350004 China; 2 Institute of Neurology, The First Affiliated Hospital of Fujian Medical University, NO. 20 Chazhong Road, Taijiang District, Fuzhou, 350004 China; 3 The First Clinical Medical College of Fujian Medical University, NO. 20 Chazhong Road, Taijiang District, Fuzhou, 350004 China

**Keywords:** Cerebral venous sinus thrombosis, CT image characteristics, Risk factor

## Abstract

**Background:**

Various computed tomography (CT) appearances of cerebral venous sinus thrombosis (CVST) were associated with different prognosis and the patients with large intracranial hematoma will have adverse outcomes, but no in-depth study of non-contrast CT image appearances was carried out. We aimed to test the hypothesis that non-contrast CT image characteristics on admission are associated with and predict the outcome of CVST at 3 months.

**Methods:**

Three hundred and six patients with CVST between 2008 and 2017 were collected. Age, sex, onset of CVST(acute, subacute, or chronic), etiology, clinical manifestations, midline shift, occluded venous sinus, location of infarction, non-contrast CT image characteristics, and the 3-month outcome were recorded. In addition, we established a non-contrast CT image-based classification and grading system to test the hypothesis; the CVST patients were classified into four grades (namely non-contrast CT image-based classification): grade I, no obvious abnormality; grade II, simple vein infarction without hemorrhage or with subarachnoid hemorrhage; grade III, cerebral venous infarction with subarachnoid hemorrhage; and grade IV, cerebral vein infarction with hematoma. All enrolled patients had received subcutaneous injections of low molecular weight heparin subcutaneous injection for 14 days. Thereafter, oral anticoagulant therapy with warfarin was continued. Patients with epilepsy were given antiepileptic drugs, and patients with cerebral herniation received decompressive craniotomy.

**Results:**

Our observational findings revealed that midline shift (> 5 mm), location of lesion (frontal lobe and temporal lobe), and cerebral venous infarction with subarachnoid or hematoma (grade III and IV) were associated with 3-month poor outcome (*p* < 0.05); the respective increased risks were 12.730 [risk ratio (RR) 12.730, 95% confidence interval (CI) 1.680–96.490, *p* = 0.014], 46.538 (RR 146.538, 95% CI 6.222–348.079, *p* = 0.000), 32.549 (RR 32.549, 95% CI 2.180–486.104, *p* = 0.012), 37.725 (RR 37.725, 95% CI 2.051–693.778, *p* = 0.015), and 93.164-fold (RR 93.164, 95% CI 11.137–779.328, *p* = 0.000). However, seizure, hemiplegia, location of occluded venous sinus (super sagittal sinus and deep venous systems), location of infarction (parietal lobe), and non-contrast CT image-based classification (I) were not correlated with the adverse outcome (*p* > 0.05).

**Conclusions:**

Our findings suggested that non-contrast CT image characteristics on admission were associated with and predict the 3-month outcome of CVST. However, the ultimate conclusions need to be confirmed by a large sample of CVST patients at multiple institutions.

## Background

Cerebral venous sinus thrombosis (CVST) is a disorder with potentially fatal consequences, which is associated with pregnancy/puerperium, infection, cancer, autoimmune diseases, thrombotic diseases, etc., and frequently affects young and middle-aged people. Despite recent advances in the management of CVST, which include subcutaneous injection of low molecular weight heparin, infusion thrombolytic therapy with urokinase through internal carotid artery(ICA), intravenous sinus contact thrombolysis, mechanical thrombectomy, balloon dilatation, and stent angioplasty, its mortality after treatment is still up to 10% [1], especially those with venous infarction and intracranial hematoma may be more prone to develop adverse outcome. It was reported that several risk factors contributing to the poor outcome of CVST were age, Glasgow Coma Scale (GCS) scores, epileptic symptoms, substantial space occupying effect of venous infarction (with or without hemorrhage) in CT/MR images, etc [2–5].

Whether side branch circulation of occluded cerebral venous sinus was effectively established is associated with the clinical outcome. The location and numbers of the occluded venous sinus, however, did not fully predict the adverse outcome. Ferro et al.’s study showed that the large parenchymal lesions in the CVST patients predicted the poor outcome, and decompressive craniotomy should be implemented as soon as possible [6]. Many neurologists might know that various CT appearances were associated with different prognosis and CVST patients with large intracranial hematoma will have adverse outcomes, but no in-depth study of non-contrast CT image appearances was carried out. Here, we analyzed the clinical images of 306 patients with CVST in our single institution, to test the hypothesis that non-contrast CT image characteristics on admission are associated with and predict the outcome of CVST at 3 months.

## Methods

All procedures performed in this observational study involving human participants were on the basis of the 1964 Helsinki declaration and approved by the ethics committee of The First Affiliated Hospital of Fujian Medical University. Informed consent was obtained from all individual participants enrolled in the study. Three hundred and six patients with CVST between 2008 and 2017 were collected. Age, sex, onset of CVST (acute, subacute, or chronic), etiology, clinical manifestations, midline shift, occluded venous sinus, location of infarction, non-contrast CT image characteristics, and the 3-month outcome were recorded. Patients were eligible for enrollment if following criteria were met: (1) the patients received brain non-contrast computed tomography (CT) scan when admitted to our institution, and CVST were confirmed by computerized tomography venography (CTV), digital subtraction angiography (DSA), or magnetic resonance imaging (MRI) with magnetic resonance venography (MRV), (2) All enrolled patients had received subcutaneous injections of low molecular weight heparin subcutaneous injection (5000 IU, per 24 h) for 14 days. Thereafter, oral anticoagulant therapy with warfarin was continued. The dose of warfarin was adjusted to achieve a target international normalized ratio (INR 2–3). Patients with epilepsy were given antiepileptic drugs, and patients with cerebral herniation received decompressive craniotomy.

**Fig. 1:**
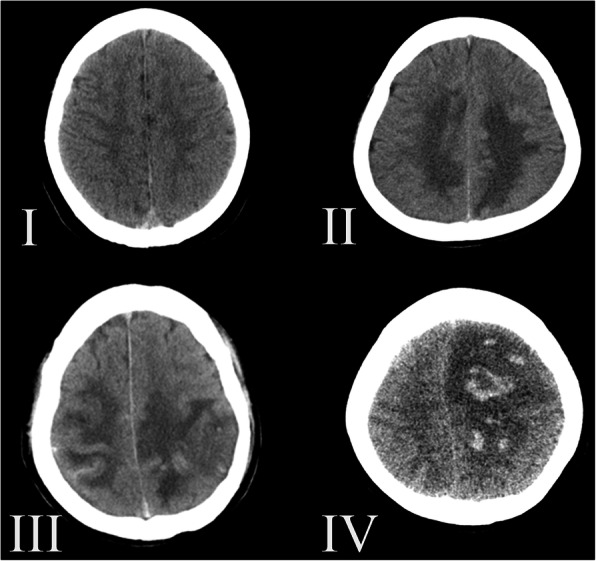
CT image-based classification and grading system: **I** no obvious abnormality (grade I); **II** simple vein infarction without hemorrhage (grade II); **III** cerebral venous infarction with subarachnoid hemorrhage (grade III); **IV** cerebral vein infarction with hematoma (grade IV)

In addition, to test the hypothesis that non-contrast CT image characteristics on admission are associated with and predict the outcome of CVST at 3 months, we established a non-contrast CT image-based classification system, which represents different stages of disease. Three hundred and six CVST patients were classified into four grades (namely non-contrast CT image-based classification): grade I, no obvious abnormality; grade II, simple vein infarction without hemorrhage or with subarachnoid hemorrhage; grade III, cerebral venous infarction with subarachnoid hemorrhage; and grade IV, cerebral vein infarction with hematoma (shown in Table 1 and Fig. 1).

**Table 1:** Non-contrast CT images-based classification for cerebral venous sinus thrombosis

Classification	Image characteristics of non-contrast CT
I	No obvious abnormality
II	Simple cerebral venous infarction
III	Cerebral venous infarction with subarachnoid hemorrhage
IV	Cerebral venous infarction with hematoma

## Statistical analysis

The statistical analyses were carried out using IBM SPSS version 22.0 (IBM Corp., Armonk, NY, USA). The significance differences in qualitative data were compared using a chi-squared test (*χ*^2^ test) or Fisher’s exact test. To avoid rejection of potentially important variables, all variables with significance level at *p* < 0.15 were included in multivariable analysis logistic regression analyses [7]. The receiver operating curve (ROC) was created using MedCalc 15.2.2 (MedCalc Software, Mariakerke, Belgium), to analyze the specificity, sensitivity, negative predictive values of non-contrast CT image-based classification, and positive predictive values of non-contrast CT image-based classification for Modified Rankin Scale (mRS).

## Results

Three hundred and six patients were included in our observational study. The basic clinical characteristics and radiological characteristics of CVST patients are shown in Tables 2 and 3. There was no statistical significance in the onset of CVST between the two groups (*p* > 0.05), which in our study was nearly acute and subacute (within 2 weeks) in 257/306 (84.0%). There were 249 (81.4%) patients in the good outcome group and 57 (18.6%) patients in the poor outcome group. The univariate analysis revealed there were significant differences detected in seizure, hemiplegia, GCS (3–8), midline shift (> 5 mm), the location of occluded venous sinus (super sagittal sinus and deep venous systems), the location of infarction (frontal lobe, parietal lobe, and temporal lobe ), no obvious abnormality (grade I), cerebral venous infarction with subarachnoid hemorrhage (grade III), and cerebral venous infarction with hematoma (grade IV) in non-contrast CT image between favorable and poor groups (*p* < 0.05). One hundred and sixty-eight patients (54.9%) were male and 138 (45.1%) were female; the gender distribution of the two groups was not statistically significant (*p* > 0.05). There were no significant statistical differences in age, sex, onset of CVST, etiology, headache, dizziness, aphasia, visual loss, the location of occluded venous sinus (inferior sagittal sinus, straight sinus, confluence of sinuses, unilateral sigmoid/transverse sinus, bilateral sigmoid/transverse sinus, unilateral jugular vein, and bilateral jugular vein), location of infarction (occipital lobe and other), and simple cerebral venous infarction (grade II) between the two groups (*p* > 0.05) (shown in Table 3).

**Table 2:** Basic clinical characteristics of patients with cerebral venous sinus thrombosis (CVST)

General information	Good outcome (*n* = 249, 81.4%)	Poor outcome (*n* = 57,18.6%)	χ2 value or Fisher’s exact	*P* value
Age			0.444	0.505
≤ 30 years	93	24		
> 30 years	156	33		
Age range (years)	15–63	14–58		
Sex			1.928	0.165
Male	132	36		
Female	117	21		
Onset of CVST			2.827	0.243
Acute	49	17		
Subacute	159	32		
Chronic	41	8		
Etiology				
Pregnancy/puerperium	18	9	0.302	0.583
Infection	9	4	1.320	0.251
Cerebral infarction	18	3	0.280	0.596
Autoimmune diseases	6	1	0.089	0.765
Thrombotic diseases	27	5	0.213	0.645
Unknown reason	33	6	0.310	0.578
Clinical manifestations			71.808	0.000
Headache, dizziness	210	45	0.970	0.325
Seizure	13	26	68.051	0.000
Hemiplegia	15	12	11.937	0.001
Aphasia	3	0	1.244	0.265
Visual loss	12	0	0.182	0.669
GCS			59.120	0.000
3–8	6	19		
9–15	243	38		
Midline shift			128.402	0.000
< 0.5	243	24		
> 0.5	6	33

**Table 3:** Radiological characteristics of patients with CVST

General information	Good outcome (*n* = 249, 81.4%)	Poor outcome (*n* = 57)	χ^2^ value	*P* value
Occluded venous sinus			10.879	0.209
Super sagittal sinus	168	48	6.261	0.012
Inferior sagittal sinus	12	3	0.019	0.889
Straight sinus	81	15	0.832	0.362
Confluence of sinuses	27	6	0.005	0.945
Unilateral sigmoid/transverse sinus	189	39	1.367	0.242
Bilateral sigmoid/ transverse sinus	63	18	0.939	0.332
Deep venous systems	18	0	4.378	0.036
Unilateral jugular vein	9	3	0.335	0.563
Bilateral jugular vein	6	0	1.401	0.237
Location of infarction			19.365	0.001
Frontal lobe	24	42	112.446	0.000
Parietal lobe	21	9	2.838	0.092
Temporal lobe	6	9	17.812	0.000
Occipital lobe	138	36	1.132	0.287
Other	3	1	3.974	0.742
Non-contrast CT image-based classification				
I	198	0	128.421	0.000
II	32	9	0.345	0.577
III	3	3	3.977	0.046
IV	16	45	152.839	0.000

All variables with significance levels at *p* < 0.15 [seizure, hemiplegia, GCS (3–8), midline shift (> 5 mm), the location of occluded venous sinus (super sagittal sinus and deep venous systems), the location of infarction (frontal lobe, parietal lobe, and temporal lobe), and cerebral venous infarction with subarachnoid hemorrhage (grade III) and cerebral venous infarction with hematoma (grade IV)] were included in multivariate logistic regression model for mRS. Our findings revealed that midline shift (> 5 mm), location of lesion (frontal lobe and temporal lobe), and cerebral venous infarction with subarachnoid hemorrhage (grade III) and cerebral venous infarction with hematoma (grade IV) were associated with 3-month poor outcome (*p* < 0.05); the respective increased risks were 12.730 [risk ratio (RR) 12.730, 95% confidence interval (CI) 1.680–96.490, *p* = 0.014], 46.538 (RR 146.538, 95% CI 6.222–348.079, *p* = 0.000), 32.549 (RR 32.549, 95% CI 2.180–486.104, *p* = 0.012), 37.725 (RR 37.725, 95% CI 2.051–693.778, *p* = 0.015), and 93.164-fold (RR 93.164, 95% CI 11.137–779.328, *p* = 0.000) (Table 4). However, seizure, hemiplegia, location of occluded venous sinus (super sagittal sinus and deep venous systems), location of infarction (parietal lobe), and non-contrast CT image-based classification (I) were not correlated with the adverse outcome (*p* > 0.05). It is noteworthy that seizure and location of occluded venous sinus (deep venous systems) exactly did not contribute to the poor outcome in our CVST patients, which is not consistent to the previous reports [5, 8].

**Table 4:** Predictors for poor outcome of CVST in multivariate model

		Unadjusted			Adjusted	
Independent Variable	OR	OR (95% CI)	*P* value	OR	OR (95% CI)	*P* value
Clinical manifestations						
Seizure	11.345	5.408–23.802	0.000	2.732	0.691–10.796	0.152
Hemiplegia	4.160	1.826–9.478	0.001	2.502	0.255–24.499	0.431
GCS (3–8)	23.625	8.934–62.475	0.000	0.210	0.30–1.476	0.117
Midline shift (> 0.5 mm)	55.687	21.202–146.266	0.000	12.730	1.680–96.490	0.014
Occluded sinus						
Super sagittal sinus	2.571	1.203–5.497	0.015	0.220	0.029–1.662	0.142
Deep venous systems	0.000	0.000	0.998	1.086	0.000	1.000
Location of infarction						
Frontal lobe	26.250	12.722–54.164	0.000	46.538	6.222–348.079	0.000
Parietal lobe	2.036	0.878–4.718	0.097	0.612	0.076–4.933	0.644
Temporal lobe	7.594	2.583–22.325	0.000	32.549	2.180–486.104	0.012
Non-contrast CT image-based classification						
I	0.000	0.000	0.994	0.000	0.000	0.996
III	4.556	0.895–23.186	0.068	37.725	2.051–693.778	0.015
IV	74.062	31.300–175.247	0.000	93.164	11.137–779.328	0.000

**Fig. 2:**
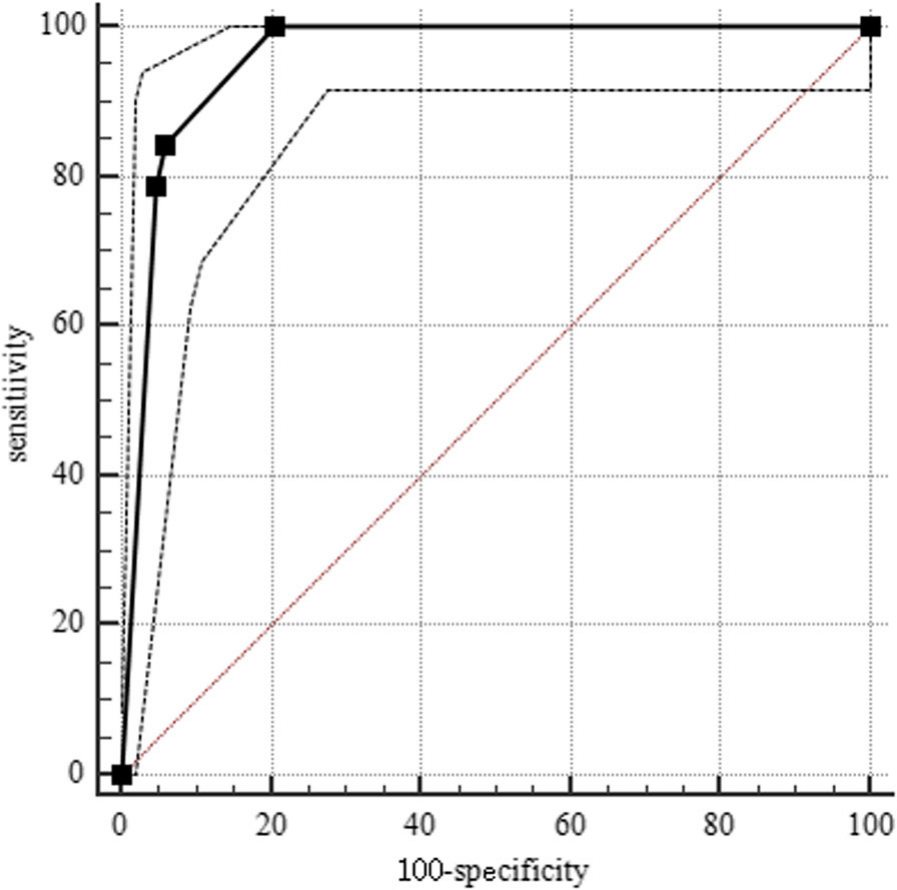
Predictive values of CT image-based classification for the 3-month Modified Rankin Scale (mRS) > 2 area under curve 0.957 (95% confidence interval [CI], 0.928–0.977; *p* < 0.00001; sensitivity, 100%; specificity, 79.52%)

**Fig. 3:**
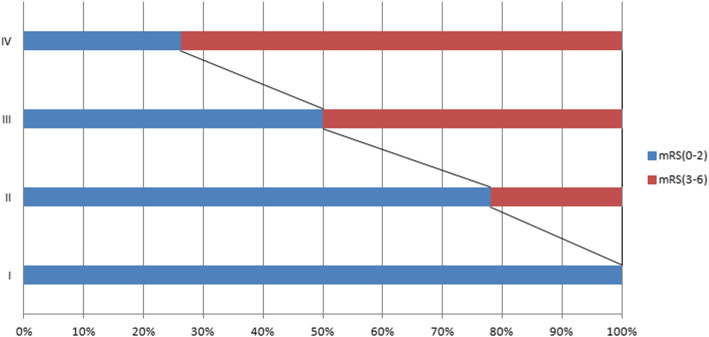
The CT image-based classification distribution of patients in the favorable and poor outcome groups

The receiver operating characteristic (ROC) curve of non-contrast CT image-based classification is shown in Fig. 2. Predictive values of the classification for the 3-month Modified Rankin Scale (mRS) > 2 area under curve were 0.957 (95% confidence interval [CI], 0.928–0.977; *p* < 0.00001; sensitivity, 100%; specificity, 79.52%). There has been an obvious trend in the distribution of patients between the favorable and poor outcome groups according to the CT image-based classification; the proportion of patients with poor outcome is more with the increasing grade of non-contrast CT image-based classification (shown in Fig. 3).

## Discussion

CVST is a fatal and disabling cerebrovascular disease, which was frequently ignored and misdiagnosed in the clinical practice because of its atypical manifestation. To date, subcutaneous injection of low-molecular-weight heparin is still the first-line treatment, because it resulted in a reduction of poor outcome and severe disability and did not promote anticoagulant-related intracranial hemorrhage. Despite recent advances in the intravascular interventional treatment, infusion thrombolytic therapy with urokinase through ICA, intravenous sinus contact thrombolysis, mechanical thrombectomy, balloon dilatation, and stent angioplasty have not significantly reduced the mortality of CVST patients. It is known that many risk factors could predict the clinical outcome of CVST. And it was reported that various CT appearances were associated with different prognosis and CVST patients with large intracranial hematoma will have adverse outcomes [6], but no in-depth study of non-contrast CT appearances related to CVST was carried out.

Here, we firstly tested the hypothesis that the non-contrast CT image characteristics of CVST was associated with and predicted the outcome of CVST at 3 months in our observational study. Our findings revealed that midline shift (> 5 mm) and location of lesion (frontal lobe and temporal lobe) were associated with 3-month poor outcome, while GCS (3–8) was not, which was reported that it was associated with the poor outcome of CVST [2, 3, 9] and maybe be related to our small number of our patients with poor outcome. Several previous reports, however, revealed that midline shift could result in poor outcome only when it was equal to or greater than 10 mm [10, 11], and cerebral herniation was the main cause of acute death in CVST patients. Emergent decompressive craniotomy should be performed [2]. Remarkably, we found that midline shift of > 5 mm would be able to cause the adverse prognosis in CVST, which was an independent risk factor which contributed to the poor outcome. It was revealed in our study that the vein infarction located in frontal lobe would be prone to cause central herniation and partially explained the mechanism why midline shift of > 5 mm would be able to cause the adverse prognosis in CVST. And it was also revealed that the vein infarction located in temporal lobe contributed to the adverse outcome, which would be prone to cause transtentorial herniation.

Interestingly, we also found that cerebral venous infarction with subarachnoid hemorrhage (grade III) and cerebral venous infarction with hematoma (grade IV) were independent risk factors which contribute to the 3-month poor outcome, while grade I (no obvious abnormality) and grade II (simple venous infarction) did not. Therefore, it was deduced that grade I in the non-contrast CT image-based classification was not associated with adverse outcome in CVST and grade II was exactly the brink of prognosis, which was the watershed to distinguish what was the risk factor. The ROC curve and the distribution of patients between the favorable and poor outcome groups according to the CT image-based classification strongly revealed that the non-contrast CT image characteristics were associated with the outcome of CVST. Non-contrast CT image-based classification was a useful tool for predicting the outcome of CVST at 3 months.

It was reported that age > 37 years and female have a much better prognosis than males [3, 12]. Nevertheless, we found that there were no significant statistical differences in age and sex between the good outcome and poor outcome groups in the univariate analysis (*p* > 0.05). Therefore, age and sex were not the risk factors contributing to poor outcome of CVST. On the contrary, we found seizure and location of occluded venous sinus (super sagittal sinus, unilateral sigmoid/transverse sinus, and deep venous systems) were not correlated with the adverse outcome (*p* > 0.05). It was reported that seizure was independently associated with supratentorial lesion; however, seizures did not correlated with the poor outcome of CVST, which is consistent with previous studies [13, 14], which cannot be fully explained by our data and not consistent with previous reports, because the number of our poor outcome group was small. Hence, the ultimate conclusions need to be further confirmed by a large sample of CVST patients.

Here, we assumed that non-contrast CT image classification was associated with the degree of sinus thrombosis of CVST, which would predict the outcome of CVST. To our best knowledge, it is the first time that the non-contrast CT classification of CVST was described. Interestingly, it was demonstrated that there has been an obvious trend in the distribution of patients with adverse/good outcome according to the CT image-based classification, and the proportion of patients with poor outcome was more with the increasing grade of non-contrast CT image-based classification.

## Limitation

Our study has its limitations: (1) It is an observational study with sample selection bias at our single institution, and the number was small. (2) The non-contrast CT image was collected on admission, which was immediate image findings and cannot reflect the dynamic changing process of CVST, especially those deteriorated after admission. (3) It is possible that the mortality of the CVST patients with grade 2–3 will be reduced with the development of drugs and interventional techniques in the future. Hence, our conclusions would be revised or be overturned.

## Conclusions

Our findings revealed that midline shift (> 5 mm), location of lesion (frontal and temporal lobe), cerebral venous infarction with subarachnoid hemorrhage (grade III), and cerebral venous infarction with hematoma (IV) were independent risk factors which contribute to the 3-month poor outcome, while no obvious abnormality (grade I) and simple cerebral venous infarction (grade II) in non-contrast CT image did not. Therefore, non-contrast CT image characteristics on admission were associated with and predict the 3-month outcome of CVST. However, the ultimate conclusions need to be confirmed by a large sample of CVST patients at multiple institutions.

## Data Availability

The datasets used and/or analyzed during the current study available from the corresponding author on reasonable request.

## Abbreviations

CT: Computed tomography
CTA: Computed tomography angiography
CTV: Computerized tomography venography
CVST: Cerebral venous sinus thrombosis
DSA: Digital subtraction angiography
GCS: Glasgow Coma Scale
ICA: Internal carotid artery
MRI: Magnetic resonance imaging
mRS: Modified Rankin Scale
MRV: Magnetic resonance venography
ROC: Receiver operating curve
RR: Risk ratio
